# Explaining the role and responsibilities of the National Anti-Coronavirus Headquarters in prevention and emergency response to pandemics in the workplace: a qualitative study on COVID-19 experience in Iran

**DOI:** 10.1186/s12913-023-09148-6

**Published:** 2023-02-09

**Authors:** Yahya Khosravi, Ali Asghar Farshad, Masoud Motalebi Gh, Mitra Faghihi, Elahe Ezati, Narmin Hassanzadeh-Rangi, Soudabeh Yarmohammadi

**Affiliations:** 1 Department of Occupational Health and Safety Engineering, School of Health, Alborz University of Medical Sciences, Karaj, Iran; 2 Research Center for Health, Safety, and Environment, Alborz University of Medical Sciences, Karaj, Iran; 3 Non-Communicable Diseases Research Center, Alborz University of Medical Sciences, Karaj, Iran; 4 Occupational Health Research Center, Iran University of Medical Sciences, Tehran, Iran; 5 Department of Health Education & Promotion, School of Public Health, Iran University of Medical Sciences, Tehran, Iran; 6 Asadabad school of Medical Sciences, Asadabad, Iran; 7 Trauma Research center, Kashan University of medical sciences, Kashan, Iran

**Keywords:** Responsibility, National Anti-Corona Headquarters, Pandemic, Workplace, Iran

## Abstract

**Background:**

In recent years, the Coronavirus disease 2019 (COVID-19) have greatly affected the safety of life and the economy. Taking rapid measures to reduce these problems requires effective and efficient decisions by various departments and headquarters in a country. The purpose of this study was to investigate the role and responsibilities of the National Anti-Corona Headquarters (NACH) in the workplace during the pandemic.

**Methods:**

This study was a qualitative study conducted using a triangulation approach. Data were obtained through semi-structured interviews with 18 participants with a purposive sampling technique as well as the review of related documents and records in response to the COVID-19 pandemic. The inductive and deductive approach was used for the content analysis of data in the Plan-Do-Check-Act (PDCA) model of the ISO45001 management system.

**Results:**

Based on the results, four themes (plan, do, check, and act) were considered as the main domains. Subthemes include understanding the needs and expectations of interested parties; specific policy-making for organizations/workplaces; leadership and organizational commitment; addressing risks and opportunities; providing resources; competence of individuals and organizations; awareness; communication; information documentation; emergency response; monitoring, analyze, and evaluate performance; management review; non-compliance and corrective action; and improvement in pandemic control.

**Conclusion:**

To ensure the effectiveness and efficiency of organizations to deal with pandemics, the NACH must implement these responsibilities and play a pivotal role in responding to pandemics and using the participation of other government agencies and society. The findings of this study can be useful from national to local levels.

## Background

The current outbreak of the Coronavirus disease 2019 (COVID-19), based in the Hubei province of the People’s Republic of China, has spread to many other countries within a few weeks [1]. On March 12, 2020, the World Health Organization (WHO) declared COVID-19 as a global disease [2]. The first confirmed case of COVID-19 in Iran was reported on February 19 [3]. Like other affected countries, Iran may not be able to escape the economic impact of the pandemic while minimizing mortality.

Identification of transmission routes and related risk factors is very important in trying to control the mode of diseases [4]. Transmission of disease through work and the workplace is one of the important factors in the spread of infectious diseases. The transmission patterns of the COVID-19 virus and asymptomatic cases can lead to high transmission rates among workers [5]. To minimize the transmission rate of this pandemic, various types of strategies and policies have been implemented in the workplace, including, social distancing which has been recommended by the WHO as a preventive measure [6].

Although, COVID-19 has not been included in occupational disease lists provided by international organizations [7], the International Labor Organization (ILO) has declared COVID-19 as a new work-related concern and recommends that all governments, countries, and regions should be prepared to intervene and control the disease [8]. The Occupational Safety & Health Administration (OSHA) stated that preventive and protective measures to control COVID-19 depend on the type of work performed and the risk of exposure to it, including the possibility of contact with infected persons and contamination of the workplace [9]. The Centers for Disease Control and Prevention (CDC) has recommended guidelines to help people identify the hazards, isolate the employees, promote hand and environmental health, implement social distancing, and encourage employers to provide health information to employees [5]. The WHO and CDC also provide guidelines for personal protective equipment to prevent COVID-19 [10, 11]. The British Health and Safety Executive (HSE) has produced extensive information on the web and standard guidelines for health and safety management as well as risk assessment in pandemics [12, 13].

The long-term solution to the COVID-19 pandemic is a globally safe vaccination program with a wide range of clinical, social, and economic benefits. However, the availability of vaccines is not enough to guarantee broad immunological protection and pandemic prevention, and control programs are still necessary [14].

The employer must adopt a new approach and new tools for efficient management of occupational health and safety. The publication of the new ISO45001 management system is an important step in occupational health and safety management. The ISO45001 management system acts as a useful tool to enable the organization to actively improve its occupational safety and health performance, regardless of size, type, and nature [15, 16]. Panahi et al. reported that industries with occupational health safety management systems had a lower incidence of COVID-19 disease than industries without these systems [17].

In early March 2020, following the decision of Iran’s Supreme National Security Council established the National Anti-Corona Headquarters (NACH) at the level of the Council of Ministers with the presence of the President. The NACH consisted of the president, ministers, and heads of governmental and public organizations. The scientific committee, economic committee, operational headquarters, and provincial headquarters worked under the NACH. The approvals of the NACH are binding for all ministries, organizations, and people of the country.

The NACH was the national measure in managing the process of combating this pandemic in Iran. Some decisions are made and implemented in this headquarters in a short- time if it was supposed to be adopted and implemented during the normal process of conviction in the country, it might take months.

During the COVID-19 pandemic, Iran’s Ministry of Health and Medical Education (MOHME) adopted extensive policy and executive measures appropriate to each age and gender group, as well as the areas of work and life of citizens, and notified other stakeholders for implementation. The MOHME, following the approvals of the NACH, took measures in two stages to combat this pandemic in the workplace. In the first stage, the preparation of workplace health protocols for jobs with essential activities was developed and communicated. As well, occupational health inspections were conducted throughout the country. All inspections were conducted by the MOHME and Iran’s Ministry of Labor on COVID-19 health protocols with a focus on small workshops [18–20].

In Iran, the most important providers of occupational health services in industries and services include occupational health committees, Behgar stations, occupational health houses and occupational health centers, and health, safety, and environment units of organizations [21].

Numerous sections of occupational health services play an important role in the process of providing health services related to the employees in Iran so the source of their effect is the integrated management of occupational health in the primary health care system of Iran; including occupational health centers in workshops, comprehensive urban and rural health centers, occupational health homes, ameliorator station, and worker and workplace health management system [21]. These centers received all health and care protocols during COVID-19 and applied them in urban and rural workplaces [19, 20].

Considering that most of the decisions of the NACH have been in the field of business and macro measures during the COVID-19 pandemic, we decided to conduct a qualitative study to examine the roles and responsibilities of the NACH to prevent and counteract the pandemic by learning from COVID-19 in the workplaces. It provides a platform for learning and sharing this experience with the WHO and other countries that have similar conditions during the COVID-19 pandemic.

## Methods

### Study design

**Fig. 1:**
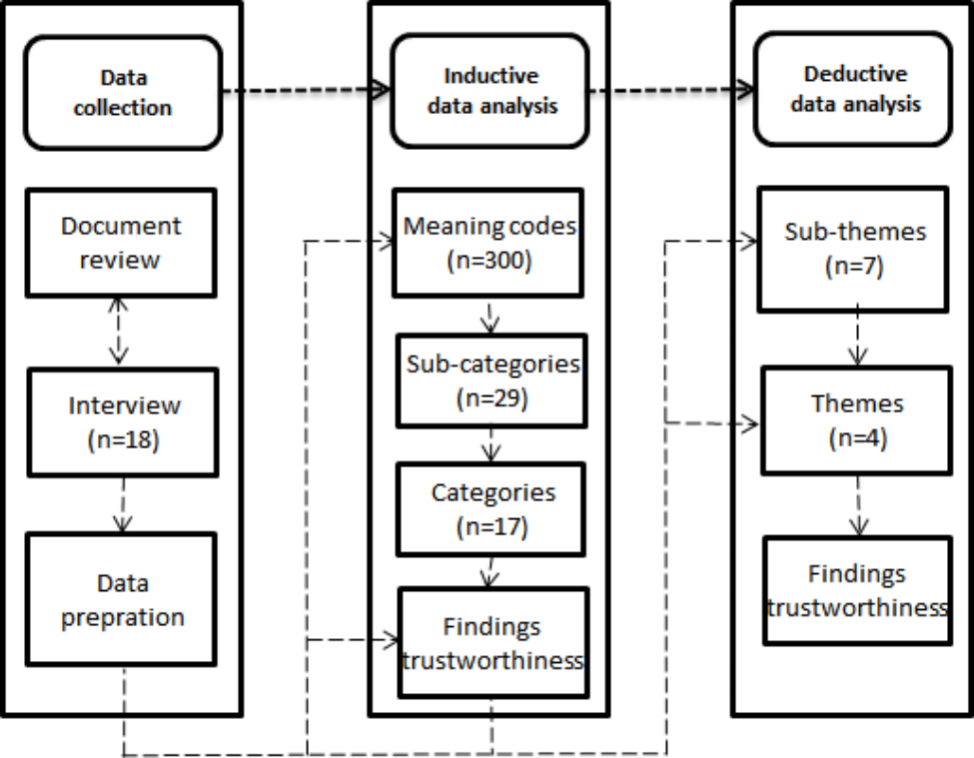
Study design

An exploratory qualitative analysis, using a triangulation approach for data collection and analysis methods, was conducted to identify the roles and responsibilities of the NACH in preventing pandemics in the workplace. Thurmond (2001) stated that” Triangulation is the combination of at least two or more theoretical perspectives, methodological approaches, data sources, investigators, or data analysis methods. The intent of using triangulation is to decrease, negate, or counterbalance the deficiency of a single strategy, thereby increasing the ability to interpret the findings” [22]. In the present study, researchers used a mixed-method approach for data collection through the lived experience of policymakers using individual semi-structured interviews and the review of related documents and records in response to the COVID-19 pandemic. The lived experience of working in the pandemic era is a unique experience that may happen in the working life of every healthcare researcher and practitioner [23]. Horton (2004) concludes that the use of semi-structured interviews provides a valuable means to allow researchers to explore their horizons [24]. Chatfield (2020) stated that well-planned secondary qualitative analysis through document review potentially reflects efficient use or reuse of resources and provides meaningful insights regarding a variety of subjects [25]. The data sources of document review and interviews were used in a complementary way. When there was a divergence between document analysis and interview findings, a decision was made with the opinion of a panel of experts consisting of practitioners and research members. The combined inductive and deductive approach was used for the content analysis of data according to the previous studies [26, 27] to triangulate the analysis method and thereby increase the power of interpretation comprehensiveness of the findings. The inductive content analysis extracted the meaning codes, sub-categories, and categories. The deductive content analysis generated the sub-themes and themes according to the Plan-Do-Check-Act (PDCA) model of the ISO45001 management system to increase the applicability of the findings of the research in workplaces [16]. Subsequently, we checked the data and findings’ trustworthiness based on different trustworthiness criteria and strategies according to the previous studies [26, 28]. The flow diagram of the study is shown in Fig. 1.

### Participants

We conducted purposive sampling to select the participants. Initially, eight cities (Tehran, Kermanshah, Qom, Kerman, Ahvaz, South Khorasan, Kashan, and Shiraz) from five regions of Iran (North, South, Central, East, and West) were identified that had the highest prevalence of COVID-19 during the study. Inclusion criteria in the study include the practitioners who have been involved in the NACH decisions. Exclusion criteria included participants unwilling to continue the interview or self-declaring of having insufficient information about the subject at the interview time. In total, 18 policymakers participated in this study; including 3 directors of infectious diseases, 3 vice chancellors for standards, 3 university presidents, 3 vice-chancellors of health affairs, 3 deputy heads of the City Health Council, and 3 mayors.

### Data collection

Data were collected in the present study, through the analysis of approvals, documents, and instructions of the NACH, including the implementation procedures of the social distancing plan in the Iranian workplaces, analysis of documents of international reference health organizations at work such as WHO, OHSHA, ILO, EU, ASH, and interviewing the policymakers.

In this study, maximum diversity was used to examine internal and external documents. All documents, the health guidelines, and procedures that were prepared in the first step against COVID-19, the analysis of the social distancing plan used in the second step in Iran, as well as foreign documents such as WHO, OSHA, ILO, and EU, ASH, which provided recommendations for the prevention of COVID-19 in the workplace, were analyzed.

For the interview, after determining the time and place of the interview, the interviewees were contacted. The interviews were audio-recorded. Interviews were conducted in a quiet place. Sampling continued until data saturation (until no new information was obtained from the data). The interviews lasted between 30 and 60 min. For the interview, an initial copy of the interview guide was prepared. The guide consisted of two parts: (1) The first part was demographic information, and (2) the second part was the main questions, including the purpose of implementing the social distance plan in the workplace, lessons learned from the implementation of this plan, infrastructure and resources and equipment required for this plan and how to monitor and evaluate it placed. After each interview, interviewees’ comments were used to complete the interview guide.

### Data analysis

To analyze the internal and external documents, instructions, and reports, the analysts carefully studied the documents, instructions, and reports and got acquainted with the basic concepts, turned them into the smallest conceptual units, and then tried to create the initial framework based on semantic similarities. The document review helped the researchers develop the semi-structured interview guide. In this way, the interview questions were based on the evidence of document review. The questions of the semi-structured interview were based on the evidence of document review.

All interviews were recorded and then transcribed by several members of the research team, the next interview was conducted after a detailed and initial analysis of each interview. The interviews were independently coded line by line by two members of the research team. In the inductive analysis step, as the analysis and continuous comparison of the extracted meaning codes progressed, their similarities and differences became clear. Finally, the meaning codes were categorized into subcategories and categories according to the previous studies [26, 27]. The first author and the corresponding author reviewed all the extracted code during a session and discussed the following themes and sub-themes, as they agreed in most cases. The extracted meaning codes were organized using MAXQDA software version 10.

In the deductive analysis step, the current study uses the PDCA model of the ISO45001 management system [16] to explain the roles and responsibilities of the NACH in the workplace, which provides a framework for workplaces to plan what they need to do. The categorized meaning codes extracted from the inductive analysis were categorized as sub-themes and themes and reported in the format of the PDCA model of the ISO45001 management system.

### Finding trustworthiness

The researchers tried to strengthen the validity of the finding as follows: (1) allocating enough time to collect data from August to November 2020 for data engagement, (2) data collection continued until no new information was obtained from the data to ensure that the data saturation occurred [29] (3) the results of the interview were return to several participants and their comments were applied to ensure that the results show a strong connection to the analyzed data [30], and 3) to ensure the accuracy of the findings, the extracted codes, sub-themes, and themes were confirmed by two peer colleagues who were not members of the research team [31], (4) the overall research findings were adopted by three colleagues who were more experienced in the qualitative study [31].

### Ethical considerations

This study was approved at the Occupational Health Research Center, Iran University of Medical Sciences, Tehran, Iran (Ethics code: IR.IUMS.REC.1400.614). In addition, informed written consent was obtained from all participants. Participants were informed that all information is as confidential and were allowed to cancel the interview.

## Results

### Participants’ characterization and themes

The age range of policymakers was between 35 and 65 years old, most participants had a master’s degree (n = 6) and a doctorate (n = 12). The work experience of policymakers ranged from 9 to 27 years, with an average of 18 years of work experience. In this study, 1500 initial meaning units/ codes were obtained from interviews and document analysis. In the inductive analysis step, 300 final meaning codes were extracted from the meaning units. The final meaning codes are categorized into 29 sub-categories and 17 categories based on similarities and differences. In the deductive analysis step, the classified codes were re-coded based on the PDCA model into 7 sub-themes and 4 themes (Table 1). The PDCA-based theme and sub-themes of roles and responsibilities of the NACH were explained as follows.

**Table 1:** Role and responsibilities of the NACH for the prevention and emergency response of the pandemic in the workplaces

Theme (deductive analysis)	Sub-theme (deductive analysis)	Category (Inductive analysis)	Sample of sub-category (Inductive analysis)
Plan	Organizational contexts	Understand the needs and expectations of interested parties to control the pandemic control	Reclassification of activities (ISIC) and occupation (ISCO) from the perspective of COVID-19
		Specific policy-making for organizations/workplaces to control the pandemic	Five specific policy packages for workplaces
	Leadership and participation	Leadership and organizational commitment to control the pandemic	The commitment of industries and services to implement partial lockdown
		Organizational roles, responsibilities, and authorities to control the pandemic	Assigning roles to organizations beyond the current laws of the country
	Planning	Address risks and opportunities to control the pandemic	Risk-based planning
		determine the objectives and plans to control the pandemic	Social distancing plan
Do	Support	Provide resources needed to control the pandemic	National mobilization and the national occupational health mobilization
		Competence of individuals and organizations to control the pandemic	Healthcare workers were selected and trained by universities of medical sciences
		Awareness of the pandemic	General awareness by provincial radio and television
		Communication during the pandemic	190 telephone-based communication system
		Information documentation of the pandemic	Uploading approvals and decisions in the rules and regulations system
	Operation	Implement plans and measures to control the pandemic	Implementation of the first and second stages of social distancing
		Emergency response in a pandemic situation	Stopping the activity of non-essential workplaces in emergencies
Check	Performance evaluation	Monitor, analyze, and evaluate performance in pandemic control	Analysis of complaints of non-compliance with health regulations
		Management review in pandemic control	Holding review meetings
Act	Improvement	Non-compliance and corrective action in the pandemic control	Referral of violations to legal authorities
		Improvement in the pandemic control	Review procedures and guidelines

### Plan domain

#### Understand the needs and expectations of interested parties to control the pandemic

The review of documents and interviews showed that the needs and expectations of the interested groups, including the organizations involved in the management of the epidemic disease, were identified through the scientific committee and operational headquarters, as well as the provincial headquarters of the NACH. The MOHME reclassified the country’s economic activities based on classification systems, including the International Standard Industrial Classification of All Economic Activities (ISIC) [32] and International Standard Classification of Occupations (ISCO) [33], from the perspective of the type of exposure, response, and control of COVID-19. One participant stated:

“Classification of organizations and workplaces should be in proportion to the status of the establishment of health management systems in the organizations/workplace, based on their type (service, industrial, administrative and educational), the size of the organization such as the number of employees, the location of organizations/workplaces in industrial estates, urban space, commercial space, outside the city, the number of clients, and the risks of the organizations/workplaces.“

#### Specific policy-making for organizations/workplaces to control the pandemic

The review of the documents showed that a total of five specific policy packages were prepared for five major occupational workplaces, including administrative affairs and public services; transportation; public places and community centers; industries and workshops; food preparation and distribution centers; and hospitals and health care service centers. Also, the NACH issued a policy letter to ministries and governmental and non-governmental organizations regarding their functions for the integrated control of COVID-19. One participant stated: “Some policies and procedures applied following the existing capacities in each country can facilitate other measures to combat the pandemic. The MOHME and the NACH have developed several policies to facilitate coping practices and to increase the resilience of workplaces and businesses against COVID-19. These policies have been adopted in two aspects: facilitating the implementation of prevention and response measures or facilitating support to deal with the critical situation caused by the COVID-19 pandemic.“

#### Leadership and organizational commitment to control the pandemic

Based on the reviewed documents, the NACH and its operational headquarters, and the corresponding provincial headquarters have been holding weekly meetings with the presence of the highest national and local officials since the beginning of the outbreak of COVID-19 in Iran. According to the documents and acknowledgments of the interviewees, the decision to a general and partial lockdown of public and economic activities during the peak of the disease indicates the commitment of the NACH and organizations to control the pandemic. Evidence extracted from interviews and documents shows at the beginning of the outbreak of COVID − 19, all industrial and service workshops committed to implementing health protocols in line with their legal and social responsibility. One of the participants stated that: “In the provinces, the governors are the representative of the government and the general managers of the organizations are the authorized representatives of the organization. Therefore, inter-sectoral cooperation at the provincial level is also a very important factor in achieving the goals and policies to combat COVID-19. The use of governance and oversight capacities that exist at the provincial level can facilitate information, training, and communication, and play a very important and vital role.“ Another participant stated that: “Attracting support at the local level can facilitate the access of the health system and the MOHME to the community at the closest level and provide services to people by the capacities that exist in the region. One participant believed that: “Social responsibility is one of the most important factors in social occasions that can be effective in controlling many social problems. Social responsibility can control many problems such as following the recommendations during the COVID-19 pandemic, whether at the individual, organizational or community levels.“ It was stated in a government circular: “the most effective asset for maintaining efficiency in any organization is manpower. Manpower is exposed to various threats. The organization loses its strengths if they lose its employees. The effects of increasing these risks are detrimental to both human resources and the organization. Reducing the effects of these risks to reduce the costs of risk, reduce the stress of risk, careful planning of manpower, empowerment of human resources and development of real capabilities and competencies of individuals are important and necessary for organizations.“

#### Organizational roles, responsibilities, and authorities to control the pandemic

The review of documents and interviews indicated that the NACH has determined and communicated the roles, responsibilities, and authorities of the ministries and organizations by their functions and by the macro policies of the control of COVID-19. The operational headquarters of the NACH was responsible for coordinating ministries and organizations in joint national and extra-organizational roles and responsibilities. The findings of document review and interviews show that during the peak of the disease in the country, the NACH gave the organizations authority beyond the country’s current laws so that they could play a more effective role in controlling the epidemic through their responsibilities. For example, they issued telecommuting permits and sealed workshops violating health protocols without common formalities. This is recorded in one of the approvals of the NACH: “One of the policies proposed by the executive organs of the country was to form a working group to prevent and combat pandemic in organizations, to determine the duties of the executive organs of the working group to prevent and deal with the pandemic in organizations.“ Elsewhere the NACH approve: “the Ministry of Defense should provide 20,000 masks (N95) and protective clothing for medical personnel daily.“

#### Address risks and opportunities and determine the objectives and plans to control the pandemic

According to the findings of the documents and interviews, the decision-making procedure of the NACH was such that the scientific committee proposed policies, objectives, and plans to control the pandemic according to the executive feedback and the trend of the pandemic in the country, and the experiences of the world. The economic committee evaluated and adjusted the economic risks and opportunities of the proposed policies, objectives, and plans. At the same time, the operational headquarters evaluated and adjusted the risks and opportunities, feasibility, and effectiveness of the proposed policies and plans. Finally, the NACH approved proposed policies, goals, and plans based on the opinions of scientific, economic, and executive experts in the relevant committees. Based on the approvals of the NACH and the interviewees’ acknowledgments, the most important risk-based decisions of the NACH were a one-month partial lockdown in March and April 2020, followed by a smart (risk-based) social distancing plan along with compliance with health protocols in public and workplaces. Also, the risk-based vaccination plan (prioritizing occupational groups with higher risk) was another plan approved by the NACH. One of the participants stated: “Given the sudden outbreak of the COVID-19 pandemic, it was necessary to prepare and provide recommendations for each group of workplaces appropriate to the type of work and its risks in the first step of dealing with the COVID-19. For this purpose, instructions were provided to the workshops and industries according to their subject.“

### Do domain

#### Provide resources needed to control the pandemic

According to the reviewed documents, the economic committee of the NACH was obliged to provide the financial resources needed for the control plans for COVID-19. The findings of the interviews and documents indicated that a part of these financial resources was allocated to implement the control plans for COVID-19, including the production of masks and disinfectants, the costs of treating patients, and the salaries of health service workers, and the other part was allocated to compensate for damages to occupational groups vulnerable to COVID-19. But most of the interviewees were not satisfied with the amount and manner of distribution of these allocated resources. Based on the interviews and review of documents, the NACH had several approved plans, including the national mobilization and the national occupational health mobilization, to compensate for the lack of human resources in the control of COVID − 19 in workplaces and public places. In mobilization plans, trained human forces from the military, government, and private organizations, trade unions, and non-governmental sectors are used to implement and monitor health protocols and mask production for the public and workplaces. One of the participants stated: “Decision-making to provide financial support to the unemployed people and to manage the pandemic was tailored to the situation in each province until the time of the interviews in this study. But the decision to be flexible in producing factories was taken at the appropriate level. Also, review of the workplace health plans, decision-making for the telecommuting programs, and the reduction of working hours was completely done.“ This is recorded in one of the approvals of the NACH: “allows the Food and Drug Administration of the MOHME, in compliance with the protection of domestic production principle, to issue a one-month license and a temporary “Iran registration code” without receiving the prescribed tariff, for import raw materials and manufacture referee, equipment, masks, clothing and protective equipment needed to prevent, treat, and control the COVID-19.“

#### Competence of individuals and organizations to control the pandemic

The NACH members consisted of the president, ministers, and heads of governmental and public organizations. The scientific committee, economic committee, operational headquarters, and provincial headquarters worked under the NACH. The review of documents and decision records showed that based on the decision and the scope of its application, the heads of the scientific, executive, economic and provincial committees of the headquarters and other competent experts and organizations are invited to decision meetings. The members of the NACH are appointed by the Supreme National Security Council and the heads of the sub-committees are appointed by the NACH. All healthcare workers and other active forces in national and occupational health mobilizations were selected and trained by 61 universities of medical sciences under the supervision of the MOHME across the country according to the phase of the disease and the ongoing plans. One of the participants said: “Each of the headquarters committees is responsible for monitoring the process of combating the COVID-19 disease and the approvals of the headquarters, and the decisions that are made in consultation with experts are also submitted to the meeting of committee chairpersons. After being approved by the meeting of the chairmen of the committees, these decisions were presented for final approval at the meeting of the NACH. So that all the approvals of the NACH will be followed for accurate implementation and monitoring of the implementation process in the operational headquarters of the NACH.“

#### Awareness of the pandemic

The review of documents and interviews indicated that awareness about COVID-19 has been given to workplaces in both general and specialized ways. General awareness has been through national and provincial radio and television, Red Crescent, chambers of guilds, sending text messages by the MOHME, the Ministry of Communications, police, and municipality, social media, and other organizations and non-governmental organizations. Special awareness has been provided with a focus on trade and small workplaces through occupational health inspectors, labor inspectors, and trade union inspectors, as well as workplace healthcare workers of industries and services, and volunteer forces in national and occupational mobilizations. One participant stated: “Public education and culture building is one of the basic pillars for the control of acute respiratory diseases such as COVID-19, which in addition to preventive policies such as inspections, enactment of coercive laws, and providing the enforcement guarantees, can have a significant impact on controlling these types of epidemics.“ Another participant stated that: “The training that each organization or different guilds give to individuals and activists in that field can be very effective. It is one of the communication channels that can be very effective in transmitting information, training, and laws related to that field of work. Also, the use of committees that exist in different organizations and guilds can be another important communication channel that can be used to transfer information, training, and laws related to that guild.“ Also one of the participants stated that: “One of the first and quickest steps taken to inform people and businesses promptly was to send text messages by the Ministry of Communications.“ Another participant believed that: “Provincial Broadcasting Centers is one of the most important factors due to regional access and broadcasting programs in the local language and dialect, which can help inform and educate in various dimensions.“ As well as, one participant stated that: “According to the COVID-19 pandemic documents in the workplace, the involvement of social affairs organizations, FATA police, and municipalities has also been important in conveying information messages to businesses.“

#### Communication during the pandemic

The ways and platforms of risk communication during the pandemic, approved by the NACH, are well documented and emphasized in the interviews as follows. The most important ways and platforms for communication about the COVID-19 pandemic in the workplace included the Guilds and Industries web-based (GIWB) system, the 4030 telephone-based system, and the 190 telephone-based system. The GIWB system was launched to reopen and operate industries and services during the pandemic, to communicate specific health protocols and monitor their implementation in workplaces, and to send public feedback to the occupational health inspectors of the MOHME. The 4030 telephone-based system was launched to advise the general public on COVID-19, including prevention, vaccination, screening, and treatment of COVID-19. The new use of the 190 telephone system in the era of COVID − 19 is to register people’s complaints about non-compliance with health protocols and how to provide health and treatment services and vaccinations. One of the participants said: “After the end of the initial quarantine in April 2020, reopening the workplaces in May, and the beginning of the second step to combat the COVID-19 pandemic, organizations and business owners are required to register in the guilds system and received a QR code”.

#### Information documentation of the pandemic

According to the review documents, all the NACH approvals are immediately uploaded to Iran’s laws and regulations system (https://qavanin.ir/) and made available to the public. All executive documents, including health protocols and inspection forms and records, were prepared and updated by the specialized working groups of the scientific and operational committees and were notified by the MOHME and the operational headquarters to the health deputy of universities of medical sciences and responsible organizations across the country. The review of documents and the interviews showed that the research project documenting the experience of prevention and control measures of COVID-19 at different levels, from the NACH to workplaces, was implemented by the MOHME with the support of the WHO office in Iran, and this work is one of its outputs. One participant stated: “the COVID-19 epidemic is a social phenomenon and dealing with it requires a social approach. In the documentation process, we must avoid the pivotal manager and the news. We move forward with an artistic look and creative ideas, and if success is achieved in this field, it can be used as a great asset both in terms of transfer of knowledge and experience and spiritual and social capital for generations to come.“

#### Implement plans and measures to control the pandemic

Based on the review of the documents, the implementation task of the NACH approvals and plans was the responsibility of the operational headquarters with the central role of the MOHME at the national level. At the provincial level, this implementation task was assigned to the provincial headquarters with the pivotal role of the medical sciences university in the province. The participants emphasized that at the time of lack of access to the vaccine, one of the most important implemented plans approved by the NACH was the smart social distancing plan. In this plan, the MOHME classified workplaces into five main business groups based on the nature of economic activities (ISIC) and related occupations (ISCO) and prepared specific health protocols for the main and sub-groups. At the beginning of the outbreak of COVID-19 in Iran, every industry or service downloads the specific health protocol of its ISIC occupational group by registering in the GIWB system and undertakes to implement the health protocols. The health supervisors, with the central role of occupational health inspectors, supervise compliance with health protocols. Occupational health inspectors, occupational health experts of industries and services, environmental health officers, Behvarzes (healthcare workers), labor inspectors, trade union officers, and other trained volunteers of the national mobilization plan monitor compliance with health protocols. One of the participants said: “during the pandemic, specific protocols for each task were sent to them and they pledged to observe the hygienic measures against COVID-19. Based on this, inspections were carried out to monitor the implementation of the protocols.“ Other participants stated that: “Due to the specialization of the subject, the MOHME was entrusted with excellent supervision over the implementation of the guidelines and health procedures against COVID-19 in the workplaces. Under the NACH, the High Committee for Supervision of Procedures was formed with the responsibility of the Deputy Minister of Health, MOHME, and membership of the Ministry of the Interior, Ministry of the Islamic Culture and Guidance, Radio and Television, Governmental Punishment Organization, Iran Chamber of Trade Unions, and relevant deputies of the MOHME.“ One of the participants stated that: “We sent instructions to the supervisors and they also informed all the employers that the main items were the employees’ health screening, identifying the high-risk people and limiting their fields of work, disinfection of the workplace, at least two-meter distance between the people, and returning to work. A list was prepared and communicated to the employers and among protection committees. Our inspectors took checklists and monitored them continuously.“

#### Emergency response in a pandemic situation

The document review and interviews show that workplaces were classified into four groups (1, 2, 3, and 4) based on their necessity of economic activity. On the other hand, all the cities of the country were coded red, orange, and yellow based on the level of emergency of the pandemic outbreak. At each emergency level, the NACH approvals allowed one or more occupational groups to operate under approved conditions. One participant describes the red emergency level as follows: “Most of the meetings are held in absentia. Training is in the form of web conferences and absentia. Communicate between employees as much as possible by observing the physical distance and making the most of human resources.“ One of the participants stated that: “The use of the mask was fully monitored. Also, the individual area was assessed at an appropriate level and the introduction of offending employees and employers was estimated at a limited level.“

### Check domain

#### Monitor, analyze, and evaluate performance and management review in pandemic control

The document review and interviews show the health supervisors, including occupational health inspectors, occupational health specialists of industries and services, environmental health officers, health workers, work inspectors, and other trained volunteers of the national mobilization plan, monitored all the workplaces’ compliance with health protocols. Health inspectors, employees, and customers were able to record the performance of workplaces regarding health protocols such as masks and physical distancing through the approved forms on different platforms including paper, telephone (such as the 4030 and 190 system), and electronic (such as the GIWB system). The performance of different occupational groups and workplaces regarding the pandemic control was reported by the MOHME to the provincial headquarters, operational headquarters, and the NACH to make decisions and take further actions. One of the participants said: “Since the weekly report on the COVID-19 outbreak was very influential in the decisions of the NACH, medical universities across the country were required to send a weekly report on the COVID-19 outbreak to the MOHME. This is recorded in one of the minutes of the NACH: “Observing intelligent spacing and reviewing and communicating procedures and instructions were fully implemented. Review of the occupational health, assessment, and control of COVID-19 risks targeted monitoring and inspection, quarantine screening, and return of employees’ plans implemented properly. But the measures were assessed by interviewees with a limited degree to support the implementation of the protocol.“

### Act domain

#### Non-compliance, corrective action, and improvement in the pandemic control

According to the documents and interviews, health inspectors with the central role of occupational health inspectors, based on pre-determined legal powers or assigned by NACH, seal workplaces that violate the social distancing plan and health protocols or refer them to legal authorities. Also, the results of the review of the documents show that the feedback received from the MOHME and operational and provincial headquarters about the outbreak of the pandemic and the implementation process of the plans were the basis for the decision of the NACH to improve the control of the pandemic. One of the participants said: “To cut off the COVID-19 transmission chain, a special health inspection program was implemented in the form of intensified supervision in all cities with the participation of all occupational health inspectors. Under the regulations, the units that did not follow the hygienic rules were dealt with decisively. Also, the necessary coordination of judicial authorities was done to deal with health violators. In the special inspection program, the maximum inspection and logistics capacity of the Deputy Minister of Health and other organizations were used. Another participant said: “After reviewing by the MOHME, the performance reports of the universities should be sent to the NACH so that these reports can be the basis for the decision of the NACH.“ One of the participants stated: “Health protocol reviews have been activated in all occupations and groups. For example, in the case of workplaces, it is determined how much time people need to stay in the workplace by calculating the area and the amount of air conditioning.“

## Discussion

In this qualitative study, an attempt was made to present the role and responsibilities of the NACH during the pandemic in the workplace. Various headquarters and organizations in the context of the pandemic, take on roles and responsibilities to prevent and combat the pandemic. One of these organizations was the NACH, which was set up in Iran due to the COVID-19 pandemic status. The roles and responsibilities of the NACH in this study are presented in the PDCA model.

Determining approvals appropriate to the workplaces during the pandemic as well as the needs and roles of each is considered one of the main responsibilities of the NACH. As an emergency occurs, organizations abandon many of their functions and retain only the major arteries of their life [34]. Organizations are determined by the type of services they provide, approvals, and roles appropriate to them, to prevent and combat the pandemics. As examples of Iran’s experiences during Covid-19, industrial organizations with a small number of workers applied to observe social distancing and many other regulations such as using masks. In addition to social distance plans and the use of masks, crowded and larger industrial organizations increased the number of work shifts and teleworking so that employees and workers could have less contact with each other. The educational organizations worked virtually and via the Internet. Also, one of the main roles of organizations was to educate and inform about COVID-19 and its prevention methods. Virtual work practices are likely to save costs, due to work structure and number of full-time employees, and reduce health risks [35]. In Iranian workplaces, occupational health organizations such as occupational health committees, Behgar stations, occupational health houses, and occupational health centers were activated in the national mobilization plan. These occupational organizations facilitated the prevention and control of the COVID-19 pandemic in the workplace. These occupational organizations already existed, and the NACH and the MOHME took advantage of these implementation platforms to control the epidemic.

Adoption and communication of executive policies to increase resilience to the pandemic in the workplace is another main responsibility of the NACH. Under the social distancing plan, most employees were forced to work remotely and work from home to control the COVID-19 outbreak. Social distancing is one of the non-pharmacological measures taken to prevent the spread of the COVID-19 pandemic. The social distancing plan seems to reduce the epidemic, but at the same time weakens the employee’s mental health. Therefore, the implementation of appropriate organizational measures to support employees and facilitate their adaptation is necessary [36].

The NACH established the facilitating policies to adapt employees to pandemic conditions. It is clear that the regulation of such policies during the pandemic was helpful, but due to the lack of a suitable platform, they were not fully implemented. For example, the executive policy of developing guidelines and electronic infrastructure was proper and complete, while the creation of a guaranteed platform for the supervision of the guild activities was applied to a limited extent. Also, empowerment and capacity building of occupation groups were only mentioned in the documents and were not fully implemented according to the opinion of the interviewees.

The NACH also enacted supportive policies such as the production of masks and other personal protection equipment (PPE), economic and insurance support, and the strengthening of transportation. But not all of these policies were fully implemented. The production of masks and PPE was fully implemented. Economic and insurance support and assistance for the less literate and rural strata has not been as expected due to the limitation of electronic registration. Also, the strengthening of public transportation has not been evaluated favorably despite being registered in documents. Considering the importance and complexity of decisions in emergencies, decision-makers should finally adopt flexible policies for pandemic control, taking into account the executive capabilities of the organization. Otherwise, these decisions create challenges for employees and other stakeholders. The study by Huremović et al. (2019) reported that the stressful situation was exacerbated by the lack of PPE during the epidemic [37]. Also, a lack of social support can affect the level of self-efficacy of workers [38]. As a result, promoting training, and safety practices and improving job support in epidemic conditions are helpful for workers’ performance [39, 40].

The NACH implemented regulatory policies for workplaces, such as rapid reporting on COVID-19 outbreaks and intensifying occupational health monitoring and inspections in the workplace with a focus on guilds. The rapid reporting COVID-19 outbreak and monitoring compliance with health protocols in workplaces were fully implemented. Also, the workplace health inspection and monitoring and the implementation of social distancing in the guilds were evaluated favorably during COVID-19.

Addressing risks and opportunities to determine and implement the strategies and plans in line with the pandemic control was considered one of the responsibilities of the NACH. The Plans based on the risk classification of occupations, such as the smart social distancing plan and the risk-based vaccination plans, were implemented in Iran. Risk classification of occupation based on function and necessity helps identify priorities and adjust possible actions such as the reactivation of one or more economic sectors during the pandemic. The analysis of risks in the workplace showed that many of the most dangerous workplaces are those that are open due to necessity [41].

In Iran, different content, methods, and platforms were used to document information, raise awareness, and communicate with the general public and workers about the pandemic. The main content was the NACH approvals and general and specific health protocols, which were well-documented and publicly available. Methods and platforms such as public media, telephone, electronic systems, social media, and workplace training were used to inform and communicate the pandemic information. Workplace safety training to combat COVID-19 can reduce the risk of COVID-19-related disease by educating staff on hygiene, face care, and cleaning measures to prevent the diffusion virus in the workplace. Leading organizations such as CDC and OSHA recommend that if possible, the pandemic guide should be industry-appropriate. The procedures are up-to-date with the CDC and OSHA information for workplace safety [42, 43]. Collecting and communicating data and generating it into helpful information is the basis of a successful program and improving the quality of that program. Tawalbeh et al. (2021) reported that the role of specialists has a positive effect on the archives and electronic documents of the COVID-19 pandemic [44].

Preparing for emergency response in the workplace during the pandemic is recognized as another responsibility of the NACH. The smart distancing plan was implemented almost in full, in which one or more occupational groups operate under approved conditions at each emergency level in each city. The structural development of the NACH vertically to the lowest parts of the workplaces through the occupational health organization of the MOHME, as well as the development of the NACH structure horizontally in all national organizations through the operational headquarters, helped improve performance in emergencies. Organizational structure complexities may reduce public health preparedness and response capacity. Assigning the organization roles for responding to a public health emergency, such as general duties, functions, responsibilities, jobs, and tasks is essential in the response process [45].

One of the important responsibilities of the NACH was to monitor and evaluate compliance with workplaces and to continuously improve the measures taken. The expanded structure of The NACH and the previous and added platforms for occupational health inspection and surveillance allowed for continuous and reliable monitoring of the response to the pandemic. The NACH updated and modified some policies and plans based on the feedback received from the pandemic trend and control plans. One of the most important tasks that any organization has after implementing an emergency response is to monitor and evaluate the performed work to determine the non-compliance and corrective action to improve the performance in the future. The framework for monitoring and evaluating strategic readiness and accountability to COVID-19 in terms of WHO consists of 8 pillars including policy and performance evaluation [46]. Monitoring and performance evaluation of the pandemic responses is a lesson learned for decision-makers to track interventions and their effects as well as identify gaps to improve the pandemic response in the future.

### Limitations

Access to policymakers was difficult and limited due to the COVID-19 situation. Another limitation of this study was the creation of similar conditions and manner of interviews, which was partially resolved by holding meetings to explain the interview method to the interviewer.

## Conclusion

According to the results of this study, the adoption and implementation of countermeasures and prevention decisions should be implemented quickly during the pandemic and reduce the organizational hierarchy to make a decision as much as possible. One of the effective measures in managing the COVID-19 pandemic in Iran was the establishment of the NACH, in which decisions were made and implemented in a short time, and all its approvals were necessary for all sectors and other organizations of the country. The PDCA-based response is the role and responsibility of pandemic headquarters that emphasized risk-based planning, support, communication infrastructure, cross-sectoral cooperation, risk-based social distancing, monitoring, and improvement. These lessons learned can be applied in other future pandemics with minimal time and cost. Finally, the knowledge created by this study can help policymakers and managers in other regional countries to be aware of the challenges posed during the pandemic for the health system and better prepare and deal with future pandemics.

## Data Availability

The dataset generated and analyzed during the current study is not publicly available due to the highly sensitive nature of interview transcript data. Publication of entire transcripts risks identifying research participants. Data used in this study is analyzed and the data is available from the corresponding author upon reasonable request.

## Abbreviations

CDC: Centers for Disease Control and Prevention
HSE: Health and Safety Executive
ILO: International Labor Organization
MOHME: Ministry of Health and Medical Education
NACH: National Anti-Corona Headquarters
OSHA: Occupational Safety & Health Administration
PPE: Personal Protective Equipment
PDCA: Plan-Do-Check-Act
COVID-19: SARS-COV-2
WHO: World Health Organization.
